# The Teeth of Our School Children

**Published:** 1895-08

**Authors:** 


### Editorial, Etc.

THE TEETH OF OUR SCHOOL CHILDREN.—J. C. McCoy, M. D., in a paper on this subject read before the Dental and Oral Surgery Section of the American Medical Association, advocates training the children in the Public Schools the proper care of the teeth. Each State Dental Association should appoint a suitable committee to arrange a manual on the subject, then induce the Educational Board of the State to adopt such a manual as a text-book to be used by teachers and taught in our Normal Schools, requiring teachers to pass an examination upon the contents of the manual and teach the subject in the schools. Out of a school of 700 pupils where Dr. McCoy distributed printed slips, "Do you cleanse your teeth with a brush every day?" Do you cleanse your teeth with a brush twice a day?" 50 cleaned their teeth twice a day, 275 used the brush sometimes, while 175 did not own a brush.

